# A High-Yield Recombinant Inactivated Whole-Virion Nasal Influenza A(H1N1)pdm09 Virus Vaccine with an Attenuated PB2 Gene

**DOI:** 10.3390/ijms26125489

**Published:** 2025-06-07

**Authors:** Seung-Eun Son, Jin-Ha Song, Ho-Won Kim, Se-Hee An, Seung-Ji Kim, Chung-Young Lee, Hyuk-Joon Kwon, Kang-Seuk Choi

**Affiliations:** 1 Laboratory of Avian Diseases, College of Veterinary Medicine, Seoul National University, Seoul 08826, Republic of Korea; arbre04@snu.ac.kr (S.-E.S.); sjh1243@snu.ac.kr (J.-H.S.); iamkhw52@snu.ac.kr (H.-W.K.); seungji910@snu.ac.kr (S.-J.K.); 2 Avian Influenza Research & Diagnostic Division, Animal and Plant Quarantine Agency, Gimcheon-si 39660, Republic of Korea; ashpri@korea.kr; 3 Department of Microbiology, School of Medicine, Kyungpook National University, Daegu 41944, Republic of Korea; cylee87@knu.ac.kr; 4 Research Institute for Veterinary Science, College of Veterinary Medicine, Seoul National University, Seoul 08826, Republic of Korea; 5 Laboratory of Poultry Medicine, Department of Farm Animal Medicine, College of Veterinary Medicine and BK21 PLUS for Veterinary Science, Seoul National University, Seoul 88026, Republic of Korea; 6 Farm Animal Clinical Training and Research Center (FACTRC), Institutes of Green Bio Science and Technology (GBST), Seoul National University, Pyeongchang 25354, Republic of Korea; 7 GeNiner Inc., Seoul 08826, Republic of Korea

**Keywords:** pandemic 2009 virus, cognate PB2, egg-propagated vaccine, high-yield, inactivated mucosal vaccine, heterosubtypic protection

## Abstract

During the 2009 H1N1 pandemic (pdm09), the poor replication of PR8-derived vaccine strains in embryonated chicken eggs (ECEs) delayed vaccine production, necessitating costly adjuvants. To improve egg-based yield, we generated PB2-substituted H1N1 strains via reverse genetics, replacing PR8 PB2 with a PB2 lacking mammalian-adaptive mutations (dtxPB2), cognate pdm09 PB2 (19PB2), or avian PB2. All PB2-substituted strains achieved over tenfold higher titers than the conventional PR8 PB2-containing strain (rGD19), with rGD19/dtxPB2 and rGD19/19PB2 exhibiting significantly higher titers and reduced murine virulence. Among these, rGD19/19PB2 produced the highest hemagglutinin (HA) yield and, when administered intranasally as a binary ethyleneimine (BEI)-inactivated whole-virion vaccine, elicited a significantly stronger broncho-alveolar IgA response than rGD19. Both rGD19 and rGD19/19PB2 provided comparable protection against a homologous H1N1 challenge, yet only rGD19/19PB2 conferred full survival protection after a lethal heterologous H3N2 challenge. These findings show that incorporation of cognate PB2 enhances H1N1 replication in ECEs and antigen yield, reduces murine virulence, and confers robust homo- and heterosubtypic protection via intranasal immunization, underscoring the promise of PB2-modified H1N1 strains as inactivated mucosal whole-virion vaccines for future vaccine development.

## 1. Introduction

Influenza A virus (IAV), a member of Orthomyxoviridae, is an enveloped, negative-sense, single-stranded RNA virus with a segmented genome [1]. Human IAV causes seasonal epidemics, causing millions of severe cases and significant mortality, along with occasional pandemics [2]. Although strain-specific vaccination is the most effective prevention, the error-prone viral RNA polymerase drives frequent mutations, causing antigenic drift that enables immune evasion [3,4]. This ongoing evolution requires regular vaccine updates based on strain surveillance and rapid manufacturing to meet global demand [4].

For decades, egg-based vaccine production has been the primary method for influenza vaccines due to its safety, cost-effectiveness, scalability, and global infrastructure, enabling rapid distribution [5,6]. Despite concerns over egg supply limitations and egg-adapted mutations potentially reducing efficacy, it remains the dominant platform [5]. In the U.S., up to 80% of influenza vaccines rely on egg-based production [7,8,9]. This method accounts for 84.5% of seasonal and 79% of pandemic influenza vaccines globally, compared to 15.5 and 21% for cell-culture-based vaccines, respectively [10]. This dominance necessitates ongoing efforts to enhance vaccine yield in embryonated chicken eggs (ECEs).

A significant challenge arises from the suboptimal growth of contemporary vaccine strains derived from human isolates in ECEs. This limitation became particularly evident during the 2009 H1N1 pandemic (pdm09), when the recommended vaccine strain A/California/04/2009 (CA/04) exhibited limited replication in ECEs, thereby delaying vaccine production and distribution [6]. The poor growth efficiency of human-derived strains in ECEs often leads to the selection of adaptive mutations in the hemagglutinin (HA) gene to enhance replication. Notably, the Q226R mutation is one of the most frequently observed and well-characterized egg-adaptive changes, promoting growth in eggs by shifting receptor binding specificity toward α2,3-linked sialo-glycans, the predominant receptor analog in avian hosts [11]. However, this mutation has been associated with diminished neutralizing activity against circulating H1N1 strains [12]. Conversely, mutations such as D225G exhibit less selectivity for avian-type receptors and have been reported to increase binding affinity to both α2,3- and α2,6-linked receptors [13]. Despite concerns regarding the potential impact on antigenicity, many currently manufactured vaccine strains retain these adaptive mutations.

To address these challenges, researchers have focused on optimizing the genetic compatibility between viral surface proteins and internal gene segments to enhance replication efficiency in ECEs. Early studies demonstrated that incorporating a homologous PB1 segment could result in higher glycoprotein density and a more balanced expression of HA and NA, thereby enhancing neutralizing ability [14,15,16]. However, although HA and PB1 of the pdm09 virus exhibited a tendency to co-incorporate into the virion, this compatibility did not necessarily result in increased viral titer, not without a compensatory mutation in PB2 [15,17]. Additionally, some approaches have involved introducing random mutations into the internal genes of the high-growth A/Puerto Rico/8/1934 (PR8) strain to enhance HA antigen yield [18]. However, conventional reverse genetics using PR8 internal genes have innate defects using the high-risk PB2 gene possessing multiple mammalian pathogenicity-related mutations (MPMs) also found in 1918-lineage H1N1 viruses, especially the E627K mutation [19].

In our previous work, we successfully engineered high-growth avian H5Nx and H9N2 vaccine strains by replacing the PR8 PB2 gene with a mammalian nonvirulent, prototypic PB2 gene of the avian H9N2 strain (A/Korea/01310/2001; 01310) [20,21]. Although substituting this PB2 alone did not enhance the replication of a Y280-like H9N2 strain, introducing minimum essential mutations (I66M, I109V, and I133V; collectively MVV) subsequently produced a more efficiently replicating recombinant virus, likely due to increased polymerase activity that better complemented the HA and NA activities of the Y280-like H9N2 virus [22]. Therefore, the PB2-mediated fine-tuning of polymerase activity can be applied to generate more efficiently replicating recombinant vaccine strains with different HA and NA activities. The evolutionary trajectory of pdm09 viruses differed from that of conventional seasonal viruses and the PR8 virus, as its genome segments originated from multiple hosts [23]. Its HA and NA genes, in particular, were subject to different evolutionary pressures than those of long-circulating seasonal strains, which impacted the N-linked glycosylation pattern and led to distinct mutations in the HA and NA that affect receptor binding and epitope variation [24]. Moreover, as shown in our previous report, pdm09 viruses exhibit a unique pattern of host-adaptive mutations, characterized by changes such as 147T, 588T, 590S, and 591R in the absence of E627K [19]. Consequently, conventional reverse genetics approaches relying solely on PR8 internal genes may prove inadequate for generating a highly replicative pdm09-based vaccine strain in ECEs, likely because they do not account for the distinct evolutionary and host-adaptation background of this novel virus.

In this study, we aimed to engineer high-growth recombinant pdm09 H1N1 vaccine strains by replacing the PR8 PB2 gene with three PB2 variants exhibiting lower mammalian pathogenicity and reduced polymerase activity. We further assessed the immune outcomes of a high-growth strains using a mucosal (intranasal) vaccination approach, as intranasal delivery is optimal for first-line defense in the respiratory tract and has been shown to induce broad, heterologous protection [25,26]. Our approach successfully generated less pathogenic, highly productive H1N1 vaccine strains with no additional mutations in the antigenic sites of HA. Notably, the incorporation of a cognate PB2 resulted in over 10-fold higher replication rates compared to the conventional H1N1 vaccine strain carrying PR8 PB2, along with increased HA antigen content. Furthermore, when formulated as a binary ethylenimine (BEI)-inactivated whole-virion mucosal vaccine, the PB2-substituted high-growth strain demonstrated enhanced immune responses and both homo- and heterosubtypic protective efficacy.

## 2. Results

### 2.1. Generation and Comparison of the Replication Efficiency of PB2-Substituted Recombinant Pandemic 2009 (pdm09) H1N1 Viruses

**Figure 1:**
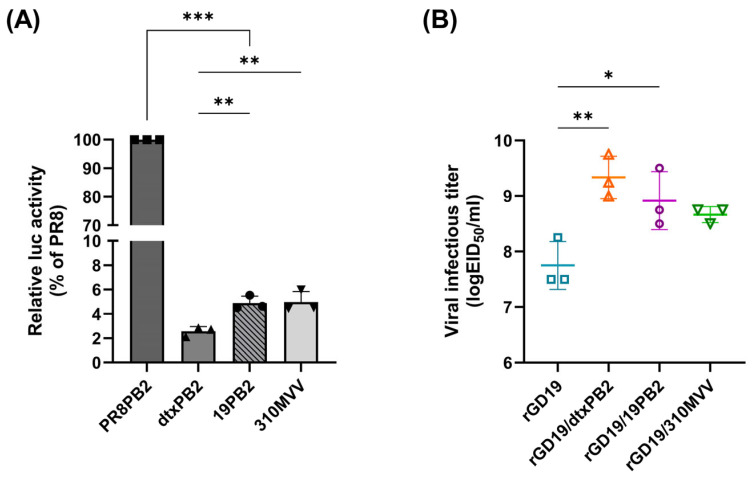
Effect of PB2-substitution in polymerase activity and viral replicative efficiency. (**A**) A comparison of the effects of PR8 PB2, 310MVV PB2, 19PB2, and dtxPB2 on the polymerase activities in 293T cells. The activities were compared using a mini-genome assay and the data were normalized as a percentage of polymerase activity of the PR8 PB2 gene. Each experiment was performed independently, and the data are presented as the mean ± SD of triplicate data from one experiment. (**B**) Viral titers (50% egg infectious dose; EID_50_) of PB2-substituted recombinant GD19 viruses. The titers were measured after passaging three times in ECEs, and data are presented as the mean ± standard deviation (SD) of three independent experiments. Statistical significance was analyzed by one-way ANOVA followed by Tukey’s multiple comparisons test (* *p* < 0.05, ** *p* < 0.01, *** *p* < 0.001).

To assess the effect of PB2 optimization in enhancing replication efficiency of recombinant H1N1 virus in embryonated chicken eggs (ECEs), we employed three PB2 variants: a prototypic avian PB2 with minimal essential mutations (310MVV), a detoxified PR8 PB2 lacking mammalian adaptive mutations (K627E, S199A, N9D, T674A, A271T, and I588A; dtxPB2), and a PB2 derived from pandemic 2009 (pdm09) H1N1 virus (19PB2). The polymerase activity of each variant was compared with that of the wild-type PR8 PB2 using a mini-genome assay (Figure 1A). All three PB2 genes (310MVV, dtxPB2, and 19PB2) exhibited over 20-fold less polymerase activities than PR8 PB2, with dtxPB2 exhibiting the lowest activity with significant differences compared to 310MVV and 19PB2.

Next, to generate PR8-derived recombinant pdm09 H1N1 vaccine strains, the HA and NA genes of contemporary H1N1 isolates representing the World Health Organization (WHO)’s recommended H1N1 vaccine strain during 2020–2021 (A/Guandong-Maonan/SWL1536/2019, GD19) were utilized. The recombinant virus representing conventional vaccine strain (rGD19) and PB2-replaced recombinant H1N1 viruses (rGD19/310MVV, rGD19/dtxPB2, and rGD19/19PB2) were generated via reverse genetics, and 50% egg infectious titers (EID_50_) were compared using allantoic fluids harvested after inoculating equal doses (100 EID_50_) of the viruses. Whole-genome sequencing confirmed no additional mutations in HA, NA, and PB2 after passages in ECEs (Figure 1B).

All PB2-substituted recombinant strains achieved higher virus titers than rGD19, surpassing a 10-fold increase (Figure 1B). Although rGD19/19PB2 (10^8.91±0.42^ EID_50_/mL) showed higher titers than rGD19/310MVV (10^8.75±0.6^ EID_50_/mL), the difference was not statistically significant. By contrast, rGD19/19PB2 and rGD19/dtxPB2 (10^9.33±0.31^ EID_50_/mL) both exhibited significantly higher titers than rGD19 and were thus selected as high-growth vaccine strains for further analysis.

### 2.2. Low Mammalian Virulence of PB2-Substituted High-Growth pdm09 Vaccine Strains

**Figure 2:**
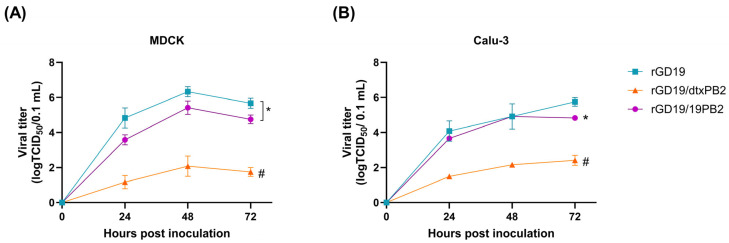
Growth kinetics of recombinant pdm09 vaccine strains in mammalian cell lines. (**A**) MDCK cells and (**B**) Calu-3 cells were inoculated with 0.01 MOI of the viruses, and supernatants were harvested 24, 48, and 72 h post-inoculation and titrated. Statistical significance was analyzed by two-way ANOVA followed by Tukey’s multiple comparisons test. (**A**) The asterisk next to the ticked bar indicates significant differences between rGD19-P and rGD19-19PB2 at all time points (* *p* < 0.01). (**B**) The asterisk indicates significant differences between rGD19-P and rGD19-cPB2 at 72 h post-inoculation (* *p* < 0.01). (**A**,**B**) The hash (#) symbols indicate significant differences between rGD19-dtxPB2 and the remaining strains at all time points (# *p* < 0.0001).

To assess the replication efficiency of the vaccine strains in different host backgrounds, we compared the growth kinetics in MDCK and Calu-3 cells (Figure 2). In contrast to the enhanced replication in ECEs, rGD19/dtxPB2 exhibited the lowest growth efficiency in both cell lines, with significant differences at all time points from rGD19 and rGD19/19PB2, consistent with its reduced polymerase activity in 293T cells. Meanwhile, rGD19/19PB2 displayed lower growth than rGD19 in MDCK cells at all time points with a significant difference (Figure 2A) yet showed similar kinetics when observed in Calu-3 cells at 24 and 48 h post-inoculation (hpi) diverging only at 72 hpi (Figure 2B).

**Figure 3:**
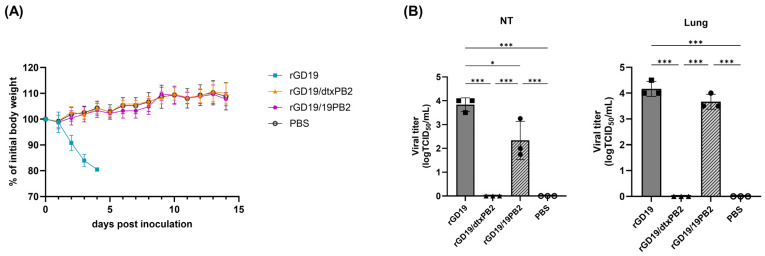
The mouse pathogenicity of recombinant pdm09 vaccine strains. Eight 6-week-old female BALB/c mice were inoculated with 10^6^ EID_50_/50 μL of either rGD19, rGD19/dtxPB2, rGD19/19PB2, or PBS. (**A**) For five mice per group, body weight change was monitored for 14 days post-inoculation, and those showing more than 20% weight loss were euthanized. (**B**) Three mice per group were euthanized 3 days post-inoculation and their lungs and nasal turbinates (NTs) were harvested for the evaluation of viral loads. The data are presented as the mean ± SD. The statistical significances were analyzed by one-way ANOVA followed by Tukey’s multiple comparisons test (* *p* < 0.05, *** *p* < 0.001).

As rGD19/19PB2 and rGD19 replicated efficiently in Calu-3 cells, we further evaluated their virulence in BALB/c mice to assess potential biosafety concerns during vaccine production [27,28]. For this purpose, equal doses (10^6^ EID_50_/50 μL) of each virus or PBS were administered intranasally, and body weight changes were monitored (Figure 3A). While rGD19 induced rapid weight loss with a 100% mortality by 4 days post-inoculation (dpi), neither rGD19/19PB2 nor rGD19/dtxPB2 caused any weight loss. Consistent with this, mice inoculated with rGD19 also displayed high viral titer both in the upper (nasal turbinates) and lower (lungs) respiratory tracts, whereas rGD19/dtxPB2 did not replicate in either site, resembling the PBS-inoculated group (Figure 3B). Notably, rGD19/19PB2 replicated to markedly lower titers in nasal tubrinates compared to rGD19, while it did efficiently in the lungs, comparable to the level of rGD19.

### 2.3. Quantitative Comparison of HA Antigen of PB2-Substituted High-Growth pdm09 Vaccine Strains

**Figure 4:**
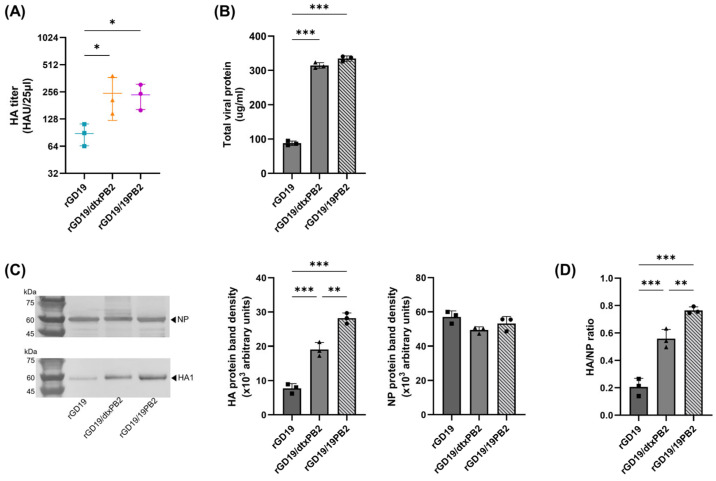
A comparison of the replicative efficiency and antigen yields of recombinant pdm09 vaccine strains in ECEs. (**A**) Hemagglutination (HA) titers of the allantoic fluids harvested after inoculating 100 EID_50_ of E2 virus stocks. The titers are shown as the geometric mean titer (GMT) ± standard deviation (SD) and indicate the average of three independent passages using 6–10 eggs. Statistical comparisons were performed on log_2_-transfomed titers using one-way ANOVA followed by Tukey’s multiple comparisons test (* *p* < 0.05). (**B**) A comparison of the total viral protein yield of semi-purified virus concentrates. The allantoic fluids harvested after inoculating an equal MOI of E2 virus stocks were concentrated under a 30% sucrose cushion, and the concentrates were quantified via a bicinchoninic acid (BCA) assay. (**C**,**D**) The band intensity of HA, NP, and the HA/NP ratio were analyzed by Western blotting followed by densitometric analysis. The densitometric analysis was performed using ImageJ software (version 1.54p), and statistical significance was analyzed by one-way ANOVA followed by Tukey’s multiple comparisons test (** *p* < 0.01, *** *p* < 0.001). The results are presented as the average of three independent experiments and bars indicate the mean ± SD. The uncropped, original Western blot image is provided in the Supplementary Figure S2.

While the infectious titer of vaccine strains represents the replicative ability of the virus, the immunogenicity and vaccine efficacy exhibit a high correlation with the amount of HA. Thus, the amount of HA contained in the vaccine strains were compared using allantoic fluids harvested after inoculating ECEs with an equal multiplicity of infection (MOI) of the viruses (100 EID_50_) (Figure 4A). The HA titers of both rGD19/19PB2 (236.8 ± 73.81) and rGD19/dtxPB2 (246.4 ± 123) were significantly higher than rGD19 (88.53 ± 24.02).

To further verify the HA content of vaccine strains, protein amounts were compared. The allantoic fluids of rGD19/19PB2, rGD19/dtxPB2, and rGD19 were semi-purified through ultracentrifugation, and viral proteins were quantified by BCA assay. The amount of total viral proteins measured by BCA assay showed no significant difference between rGD19/dtxPB2 and rGD19/19PB2, while both were over 3-fold higher than rGD19 (Figure 4B). In addition, we further compared the amount of antigenic proteins using Western blotting (Figure 4C,D). The HA and NP bands were visualized using the swine H1N1 HA monoclonal antibody and influenza NP polyclonal antibody, and the band intensities were analyzed by densitometric analysis. rGD19/19PB2 exhibited a significantly higher HA protein band intensity compared to rGD19 (Figure 4C). While both rGD19/dtxPB2 and rGD19/19PB2 exhibited higher HA/NP ratios compared to rGD19, rGD19/19PB2 showed a significantly increased ratio compared to the other viruses (Figure 4D). These results suggest that a highly infectious titer does not necessarily correlate with a high antigen amount, as witnessed by rGD19/dtxPB2. While both rGD19/dtxPB2 and rGD19/19PB2 replicated in ECEs with high efficiency, there was a stark difference in the speculated amount of HA antigen content, as well as HA density per virus particle. Overall, rGD19/19PB2 was considered more suitable for the high-growth H1N1 vaccine strain for further analysis.

### 2.4. Comparable Serological Immune Response After the Intramuscular Administration of BEI-Inactivated rGD19/19PB2 Vaccine

Although in vitro assessments provide an initial indication of vaccine potency, they do not necessarily predict the in vivo immune response magnitude or protective efficacy. To address this, we performed a mouse model study to evaluate and compare the serological immune responses of the inactivated rGD19 and rGD19/19PB2 vaccines. Specifically, ECEs were inoculated with each virus at an equal MOI (100 EID_50_), and the harvested allantoic fluids were inactivated using binary ethylenimine (BEI). Subsequent HA assays confirmed that rGD19/19PB2 contained a higher HA titer per equal volume of allantoic fluid compared to rGD19, indicating that an equivalent volume of rGD19/19PB2 delivers a higher antigen dose.

**Figure 5:**
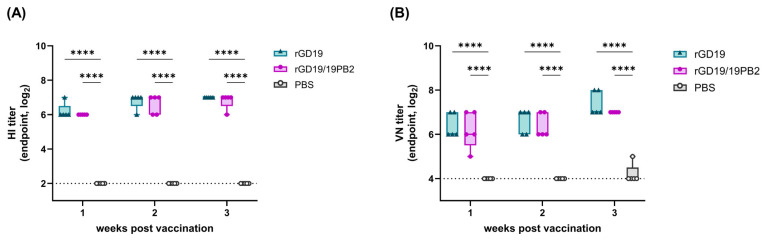
Hemagglutination inhibition (HI) and viral neutralization (VN) titers in mice intramuscularly vaccinated with BEI-inactivated pdm09 vaccine strains. BALB/c mice (*n* = 5) were immunized intramuscularly with 100 µL of clarified allantoic fluid (50 µL per hindlimb) containing BEI-inactivated whole-virus vaccine or PBS, each formulated with MF59-adjuvant. (**A**) The hemagglutination inhibition (HI) titers and (**B**) the viral neutralization (VN) titers of 2-fold serially diluted serum samples against GD19 virus antigen. Blood samples were collected every week after the vaccination. The endpoint titers were defined as the highest serum dilution with the complete inhibition of viral growth. The data are presented as box-and-whisker plots with median, interquartile range, and minimum-to-maximum values. The dotted lines indicate the limit of detection. Statistical significance was analyzed by one-way ANOVA followed by Tukey’s multiple comparisons test (**** *p* < 0.0001).

Both BEI-inactivated vaccine strains were then formulated with MF59 and administered intramuscularly (IM) to mice. Serum samples were harvested every week for 3 weeks post-vaccination (wpv), and hemagglutination inhibition (HI) and viral neutralization (VN) titers were measured (Figure 5). All the vaccinated groups exhibited significantly higher HI and VN titers than the PBS group, with an apparent increase in antibody level even at 1 wpv with a gradual increase over time. However, rGD19/19PB2 did not exhibit a significant difference from rGD19 in HI and VN titers at any time point, suggesting comparable serological immune responses between the two strains under these conditions.

### 2.5. Enhanced Humoral Immune Responses After the Intranasal Administration of BEI-Inactivated rGD19/19PB2 Vaccine

Intranasal immunization induces broad immune responses against the influenza virus by eliciting both systemic and local immunity at the primary site of infection. To elucidate the differences in immune outcomes between the two vaccine strains from a broader perspective and to characterize the immune profiles of mucosal vaccination with inactivated whole-virus vaccines, we further compared the immune responses of intranasally administered BEI-inactivated rGD19 and rGD19/19PB2 vaccines in BALB/c mice. For consistency, the vaccines were prepared under the same conditions as the IM formulations, and subsequent HA assays confirmed that rGD19/19PB2 exhibited a higher HA titer per equal volume of allantoic fluid compared to rGD19.

**Figure 6:**
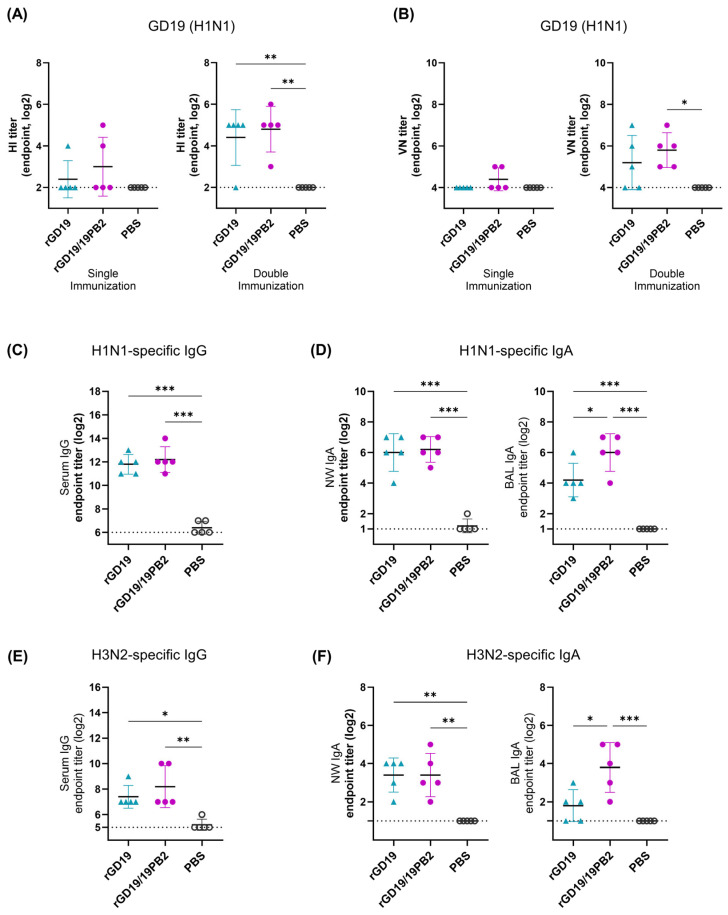
A comparison of humoral immune responses induced by rGD19/19PB2 and rGD19 vaccines against the GD19 (H1N1) and A/Aichi (H3N2) antigens. BALB/c mice (*n* = 5/group) were intranasally inoculated with 50 µL of clarified allantoic fluid containing BEI-inactivated whole-virus vaccine or equal volume of PBS, either once or twice at 2-week intervals. (**A**) The hemagglutination inhibition (HI) titers and (**B**) the viral neutralization (VN) titers of 2-fold serially diluted serum samples against GD19 virus antigen. The endpoint titers were defined as the highest serum dilution with the complete inhibition of viral growth. (**C**,**D**) H1N1-specific IgG and IgA ELISA using purified GD19 virus. (**E**,**F**) H3N2-specific IgG and IgA ELISA using purified Aichi/68 virus (H3N2). (**C**,**E**) Two-fold-diluted serum samples were incubated with anti-mouse IgG antibodies, and (**D**,**F**) two-fold-diluted NW and BAL samples were incubated with anti-mouse IgA antibodies. The optical density (OD) was detected at 450 nm, and the data are presented as endpoint titer determined as the reciprocal titers of the highest sample dilutions that yielded an absorbance greater than twice the mean of the blank for IgG ELISA and mean + 2SD of the blank for IgA ELISA. The data are presented as the mean ± SD and the dotted lines indicate the limit of detection. Statistical significance was analyzed by one-way ANOVA followed by Tukey’s multiple comparisons test (* *p* < 0.05, ** *p* < 0.01, *** *p* < 0.001).

BALB/c mice received one or two IN immunizations at two-week intervals, followed by a challenge with 10 MLD_50_ of the homologous wild-type GD19 virus two weeks after the final vaccination. Serum samples were collected 2 wpv for HI and VN tests against a homologous H1N1 antigen to determine humoral immunity (Figure 6A,B). Both vaccine strains exhibited significantly higher HI titers compared to the PBS group when receiving double immunization. Higher HI antibody titers compared to rGD19 were observed in mice vaccinated with a single or double dose of rGD19/19PB2; however, no significant differences were observed (Figure 6A). A viral neutralization (VN) test exhibited a similar tendency, with rGD19/19PB2 exhibiting higher VN antibodies in both vaccine regimens without significant differences between the two vaccine groups. Notably, only the group receiving immunization twice with rGD19/19PB2 produced VN titers significantly above those of the PBS group, whereas rGD19 did not (Figure 6B).

To further characterize systemic and mucosal antibody responses, we measured virus-specific IgG and IgA levels using an ELISA with GD19 antigen (Figure 6C,D). Both vaccine strains induced significantly higher levels of serum IgG after double immunization, while the difference between the two vaccine groups was not apparent (Figure 6C). Similarly, IgA levels of the nasal wash (NW) specimen increased in both vaccine groups with no difference between each other. By contrast, IgA levels of bronchoalveolar lavage (BAL) fluids from mice vaccinated with rGD19/19PB2 contained significantly higher IgA levels than those of rGD19, suggesting enhanced local mucosal immunity in the lower respiratory tract (Figure 6D).

Additionally, the presence of cross-reactive antibodies against the A/Aichi/2/1968 (H3N2) strain (Aichi/68) were measured. Although HI and VN titers specific to Aichi/68 were undetectable, the serum IgG levels detected by H3N2-coated ELISA showed increased H3N2-specific IgG levels in both vaccinated serum samples, with a significant difference compared to the PBS-inoculated group (Figure 6E). Similarly, the levels of H3N2-specific IgA were also significantly elevated in NW samples in both vaccine groups compared to the PBS control (Figure 6F). However, no significant differences were observed regarding the serum IgG level and NW IgA levels between the two vaccine groups. In contrast, rGD19/19PB2 induced significantly higher H3N2-specific IgA levels in BAL samples than either rGD19 or PBS, paralleling the increase observed for H1N1-specific IgA (Figure 6D,F).

### 2.6. Homo- and Heterosubtypic Protection Efficacy of the Intranasally Administered BEI-Inactivated rGD19/19PB2 Vaccine

**Figure 7:**
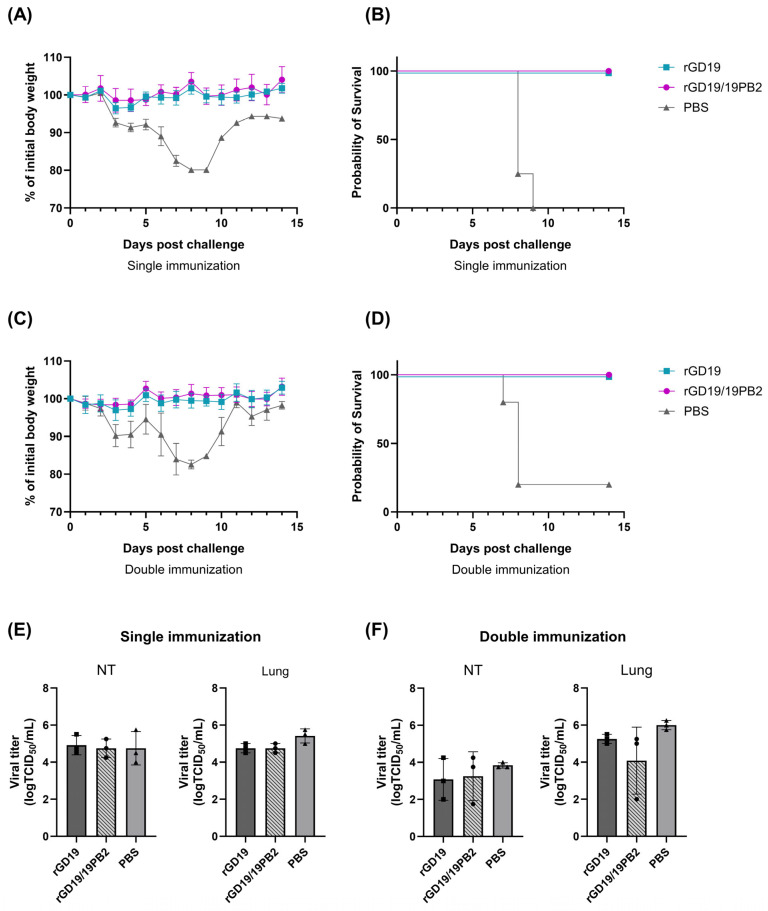
Protective efficacy of recombinant pdm09 vaccine strains against homologous (H1N1) challenge. BALB/c mice (*n* = 5/group) were intranasally inoculated with 50 µL of clarified allantoic fluid containing BEI-inactivated whole-virus vaccine or equal volume of PBS either once or twice at 2-week intervals. Two weeks after a single dose (**A**,**B**) or double dose (**C**,**D**) of the vaccines, mice were challenged with 10 MLD_50_ of homologous (H1N1) strain, and body weight change and survival rate of mice were monitored for fourteen days. Mice showing more than 20% weight loss were euthanized. (**E**,**F**) Evaluation of viral loads in the nasal turbinates (NTs) and lungs of mice 3 days post-challenge after a single (**E**) or double (**F**) dose of vaccination (*n* = 3/group). Data are presented as mean ± SD, and statistical significances were analyzed by one-way ANOVA followed by Tukey’s multiple comparisons test.

The homosubtypic protective efficacy of the inactivated mucosal vaccines was evaluated through single and double intranasal immunization with BEI-inactivated rGD19 and rGD19/19PB2 vaccines. Mice were inoculated intranasally with either the vaccines prepared as previously described or PBS once or twice at two-week intervals. Two weeks after the final vaccination, the mice were challenged with 10 MLD_50_ of GD19 virus (H1N1) intranasally, and body weight change and survival were monitored for 14 days post-challenge (Figure 7).

When challenged with a lethal dose of homologous GD19 H1N1 virus (10MLD_50_), both vaccine strains conferred complete protection in mice that received either a single (Figure 7A,B) or a double dose (Figure 7C,D). In contrast, the PBS groups exhibited 100 and 80% mortalities, respectively. In addition, the viral loads in the lungs and nasal turbinates (NTs) of mice 3 days post-challenge were evaluated (Figure 7E,F). Despite the absence of clinical signs and 100% survival in both vaccinated groups, virus replication was detected in the respiratory tracts. The titers in the nasal turbinates and lungs of single-vaccinated mice were comparable between the vaccine and PBS groups, with no significant differences between the three groups (Figure 7E). Similarly, virus titers in the nasal turbinates and lungs of double-vaccinated mice showed no significant differences between the groups (Figure 7F). Notably, mice receiving the double dose of rGD19/19PB2 vaccine exhibited the lowest viral titers in the lungs, although this difference did not reach statistical significance. Thus, neither vaccine achieved sterilizing immunity, although both markedly prevented weight loss and mortality.

**Figure 8:**
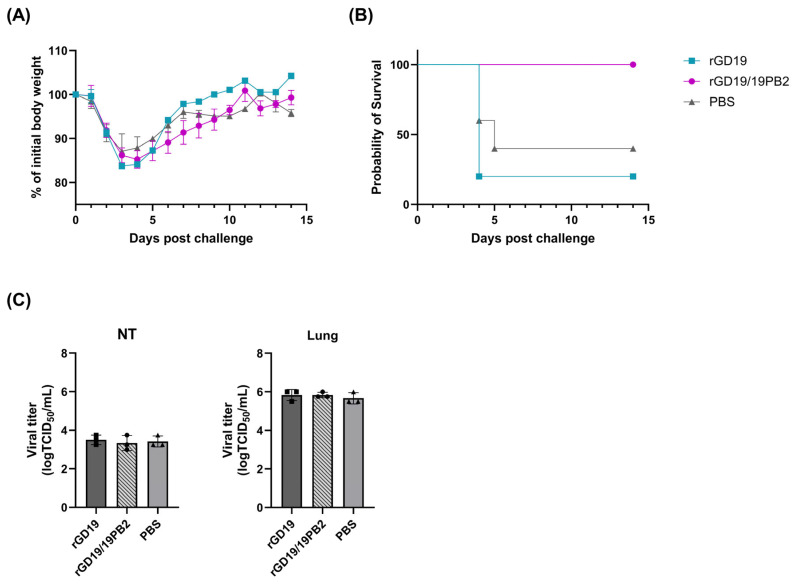
Protective efficacy of recombinant pdm09 vaccine strains against heterologous (H3N2) challenge. BALB/c mice (*n* = 5/group) were intranasally inoculated with 50 µL of clarified allantoic fluid containing BEI-inactivated whole-virus vaccines or an equal volume of PBS twice at 2-week intervals. Two weeks after the final dose, mice were challenged with 1 MLD_50_ of heterologous (H3N2) strain. (**A**,**B**) Body weight change and survival rates were monitored for 14 days post-challenge, and those showing more than 20% weight loss were euthanized (*n* = 5/group). (**C**) Evaluation of viral loads in the nasal turbinates (NTs) and lungs of mice 3 days post-challenge after a double dose of vaccination (*n* = 3/group). Data are presented as mean ± SD and statistical significances were analyzed by one-way ANOVA followed by Tukey’s multiple comparisons test.

To further distinguish the protective efficacy of the two vaccine strains and assess the efficacy of the inactivated mucosal whole-virus vaccine against a heterologous virus challenge, mice that were intranasally vaccinated twice were challenged with 1 MLD_50_ of the heterologous H3N2 strain Aichi/68 two weeks after the final vaccination. Although neither vaccine prevented body weight loss (Figure 8A), the rGD19/19PB2 vaccine provided full survival protection against mortality, in contrast to the rGD19 vaccine, which resulted in 80% mortality (Figure 8B). Similar to the homosubtypic challenge, the viral loads in the lungs and NT three days post-challenge were comparable among groups (Figure 8C).

### 2.7. Evaluation of Cellular Immune Responses

In addition to mucosal immunity, cell-mediated immunity may play a crucial role in the cross-subtype protection conferred by the intranasal administration of an inactivated whole virus vaccine. Therefore, we compared the level of cellular immunity elicited by the two vaccine strains. Lymphocytes from the lungs and spleens of mice were collected five days post-challenge and analyzed by flow cytometry following ex vivo stimulation with the homologous antigen (Aichi/68) (Figure S1). No significant differences between the CD4+ and CD8+ T-cell frequencies in the lung and spleens were observed among groups. However, rGD19/19PB2-vaccinated mice showed a significant increase in interferon-gamma (IFN-γ) secreting CD4+ T cells in the lungs and CD8+ T cells in the spleen compared to the PBS controls (Figure S1).

In summary, these results demonstrate that the rGD19/19PB2 vaccine confers superior protective efficacy compared to the rGD19 vaccine, particularly under heterosubtypic challenge conditions, thereby validating the inactivated mucosal vaccination approach. Although both rGD19/19PB2 and rGD19 induce comparable systemic and mucosal H3N2-specific antibody responses, the higher BAL IgA levels and elevated IFN-γ-secreting T cells in rGD19/19PB2-vaccinated mice most likely underlie the enhanced cross-subtype protection against Aichi/68.

## 3. Discussion

Maintaining a functional balance between the influenza A virus (IAV) surface glycoproteins hemagglutinin (HA) and neuraminidase (NA) is crucial for viral fitness and survival. During host adaptation or immune evasion, mutations that alter the activity of one surface protein are often accompanied by compensatory changes in the other, thereby preserving functional equilibrium [29,30]. Moreover, the evolution of IAV surface proteins is closely intertwined with that of internal proteins, as polymerase interactions play a pivotal role in efficient viral replication [31,32]. The standard reverse genetics platform employs the internal gene segments of the PR8 strain, which exhibits a relatively high genetic correlation to conventional seasonal flu viruses derived from the 1918 H1N1 lineage. Although this approach has been successful in generating recombinant vaccine strains against seasonal influenza, the PR8 internal genome has proven ineffective in producing the pdm09 vaccine strain [6].

The evolutionary trajectories of pdm09 and conventional seasonal influenza viruses differ significantly, as demonstrated by distinct patterns of mammalian adaptation mutations (MPMs) within the PB2 gene. The PB2 gene of pdm09 is characterized by the sequential acquisition of the mutations G590S, I147T, Q591R, T271A, and A588T [19]. While G590S and I147T are of avian origin, the later emergence of the swine-origin Q591R mutation suggests that the pdm09 virus initially adapted to its first mammalian host using a weaker mutation rather than the more potent E627K. This is further supported by the marked differences in polymerase activity observed when replacing PR8 PB2 with dtxPB2, 19PB2, or 310MVV in mammalian cells—a variation likely attributable to the presence or absence of E627K. The higher infectivity of recombinant GD19 viruses with lower PB2 activity suggests that it is conceivable that a PB2 gene exhibiting lower polymerase activity in mammalian cells may be better optimized for the avian host environment of ECEs, thereby enhancing GD19 replication (Figure 1).

The absence of mammalian-adaptive markers such as E627K in 310PB2, dtxPB2, and 19PB2 suggests that these PB2 variants may engage more effectively with avian host factors, thereby better harnessing the avian cellular machinery for viral replication and leading to increased replicative activity in ECEs. In support of this, increased viral growth in ECEs following avian signature mutation in PB2 was also observed in a previous study that discussed the effect of N701D mutation [15]. However, despite exclusively avian-specific adaptations and high infectious titer, the rGD19/dtxPB2 virus exhibited a relatively lower HA yield and HA/NP ratio compared to rGD19/19PB2. Although dtxPB2 may enhance vRNP replication in eggs, it could simultaneously impair the coordinated translation or trafficking of GD19 HA/NA, yielding fully infectious virions with fewer surface glycoproteins. Also, relatively lower packaging signal compatibility between dtxPB2 and GD19 HA/NA compared to 19PB2 might result in less efficient HA expression and incorporation during virion assembly. In contrast, the increase in the HA/NP ratio per virion without a corresponding increase in viral titer was an unexpected finding in rGD19/19PB2 (Figure 4D). A higher HA/NP ratio has been associated with filamentous virion morphology and reduced virulence due to impaired cell-to-cell transmission compared to the globular virion form. This phenomenon has been linked to the functions of NP and M1 proteins [33,34]. However, as we did not examine the ultrastructure of rGD19/19PB2, the unexpected shift in the HA/NP ratio following 19PB2 replacement requires further investigation.

Building on these insights, our study is the first to validate the use of a cognate PB2 in the development of a pdm09 vaccine strain, extending our earlier successes with highly productive recombinant avian influenza vaccine strains in ECEs [20,22]. In addition, although the detailed titers and sequence data of commercial strains remain unpublished or proprietary, many are produced following extensive egg passages. Our approach, however, yields a highly productive vaccine strain without additional passages or adaptive mutations, offering a new avenue for developing high-growth vaccine strains suitable for efficient egg-based production.

One limitation of this study is that we focused solely on the GD19 virus, which was prevalent from 2019 to 2021. Further evaluation with multiple H1N1 strains, including more recent isolates, is therefore warranted to confirm broader applicability. In addition, validation with the H3N2 and influenza B strains would reveal whether the increased HA yield reflects an intrinsic property of the pdm09 PB2 or a favorable interaction between PB2 and the surface glycoproteins. Comparative rescues that swap the cognate PB2 segments of H3N2 and influenza B with the pdm09 PB2 will be instrumental in determining the generalizability of our strategy. Moreover, as current commercial vaccines rely on 6:2 or 5:3 reassortants with PR8 generated by both classical reassortment and reverse genetics, additional studies under a classical reassortment setting are necessary to determine whether this approach can be applied to commercial vaccine production [35,36]. Additionally, we used crude allantoic fluids for our overall evaluation, providing only an initial insight into the potential yield benefits of rGD19/19PB2. Since unpurified allantoic fluid is not used for human immunization and typical manufacturing involves multiple downstream steps, including clarification, sucrose-gradient or chromatographic purification, detergent splitting, and potency standardization by single-radial immunodiffusion, further research incorporating these procedures is needed to determine whether the approximately three-fold increase in HA yield observed here (Figure 4) translates into tangible economic and practical advantages in a commercial setting. If that gain is conserved, the overall yield would increase from the published egg-based range of 0.5–2.0 to 1.5–6 doses per egg, provided that stability testing under refrigerated, accelerated temperature and repeated freeze–thaw conditions confirms that the PB2-modified antigen retains HA potency and conformational integrity comparable to conventional strains [37]. Defining the minimum protective dose through dose–response studies will then indicate whether the higher per-egg HA can be leveraged for dose-sparing strategies, an advantage that becomes critical when vaccine supplies are stretched during a pandemic.

Our findings reveal that rGD19 exhibits markedly high virulence in BALB/c mice, comparable to the wild-type GD19 strain. While the PR8 strain commonly used in vaccine production is considered safe for humans, potential biosafety issues and the remote possibility of reassortment warrant attention if accidental exposure occurs, particularly given rGD19’s robust replication in mammalian cells. Moreover, studying the polymerase gene constellations that underlie attenuation in mice can inform our broader understanding of influenza pathogenesis and guide the design of live-attenuated influenza vaccine (LAIV), where in vivo virulence is directly relevant. An egg-based LAIV platform is especially attractive for resource-limited settings, because it leverages existing egg infrastructure, bypasses costly inactivation steps, and does not require adjuvants. Although future studies must confirm genetic stability after serial passage and demonstrate safety and immunogenicity in larger animal models, the PB2-attenuated backbone could provide a practical, low-cost route to locally manufactured influenza vaccines for countries with limited technical and financial resources.

As expected, rGD19/dtxPB2 exhibited significantly reduced viral titers in mammalian cells and showed no virulence, likely due to the removal of MPMs in dtxPB2. Notably, rGD19/19PB2 also exhibited an attenuated phenotype, with no clinical signs or gross lesions indicative of mouse virulence. Although PB2 G590S and Q591R can partially compensate for the lack of 627K by supporting replication under mammalian conditions, their effects are considered less potent than those of 627K, contributing to reduced immune evasion and overall pathogenicity [38]. Likewise, the PB2-588I mutation has been linked to enhanced virulence via stronger mitochondrial antiviral signaling protein (MAVS) binding and IFN-β suppression, so retaining 588T in 19PB2 may further limit immune evasion. Nevertheless, despite its reduced virulence in mice, rGD19/19PB2 retained the ability to replicate in Calu-3 cells, as well as in the nasal turbinates and lungs of mice. The reduced replication of rGD19/19PB2 in the nasal turbinates compared to lungs is unlikely to stem from receptor tropism, because both vaccine strains share an identical HA/NA constellation and therefore bind α2,6- and α2,3-linked sialic acids equivalently. Instead, we attribute the poor replication in the upper airway to temperature-dependent polymerase efficiency governed by the 627E of 19PB2, which performs sub-optimally at the lower temperature of the upper respiratory tract (33 °C) [39]. Despite this limitation, the newly acquired PB2 mutations (V344M, I354L, S453T, D195N, and D81N) appear to compensate and facilitate replication in mammalian cells [19,38,40,41,42,43,44]. Furthermore, many of these novel PB2 mutations appear to interact with mutations in PB1 and PA, which were also found in PR8 PB1 and PA [42,45]. However, unlike rGD19/19PB2, the wild-type GD19 virus appears to maintain heightened virulence in mice, which may be the effect of fully compatible polymerase genes and additional PA mutations (e.g., L295P and P224S) that have been implicated in increasing virulence in pdm09 H1N1 mouse models [46,47].

Under identical propagation conditions, rGD19/19PB2 produced significantly higher viral titers and greater HA yields compared to rGD19 (Figure 1 and Figure 4). However, the difference in dose, which is attributable to the enhanced propagation capacity of rGD19/19PB2, did not result in significantly higher systemic antibody titers when administered intramuscularly in mice. Both vaccine strains induced serum antibody levels indicative of protection, as formally determined in a mouse model [48,49,50,51]. However, the lack of significant differences in antibody titers between the two strains at all time points and the modest rise from week 1 to week 3 was unexpected. The squalene-based MF59-like adjuvant elicited a strong early antibody response to both rGD19 and rGD19/19PB2 vaccines at 1-week post-vaccination. One plausible explanation is an adjuvant-driven ceiling effect. MF59 rapidly recruits and saturates antigen-presenting cells once a threshold amount of HA is present, limiting further expansion of antibody-secreting cells [52]. The subsequent plateau from weeks 1 to 3 may reflect germinal-center contraction as antigen display and T-follicular-helper support wane [53]. Booster immunization or antibody-avidity assays could determine whether rGD19/19PB2 ultimately provides superior antibody quality, even when peak titers appear similar. It is also noteworthy that, in avian studies where oil-emulsion vaccines (e.g., ISA-70) and a higher viral dose (e.g., 10⁹ EID_50_/mL) are used, HI and VN titers often reach approximately 9–10 (log_2_) by 3 wpv in SPF chickens. Although those data are not directly comparable, they illustrate the influence that different adjuvants and higher antigen doses can have on antibody responses. The relatively modest increases in HI and VN titers at 3 wpv may therefore be attributable to distinctions in the adjuvant type, antigen content, or inherent immunological differences between species [48,54,55].

As similar systemic antibody levels were observed among mice intramuscularly vaccinated with rGD19 and rGD19/19PB2, one might infer that the rGD19/19PB2 vaccine requires a higher antigen dose to achieve comparable immune responses. However, the intranasal inoculation of these inactivated vaccines revealed significant differences between the two strains. Despite containing only pdm09 H1N1 antigens, rGD19/19PB2 conferred complete protection (100% survival) against a heterologous H3N2 challenge when intranasally administered, whereas rGD19 allowed 80% mortality. This unexpected outcome prompted a closer comparison of systemic and mucosal antibody responses. Serum antibody titers did not differ significantly between the two vaccine groups, yet rGD19/19PB2 elicited substantially higher secretory IgA (sIgA) in bronchoalveolar lavage (BAL) fluid. Both H1N1- and H3N2-specific IgA levels were elevated, but only the H3N2 challenge demonstrated a clear difference in protective outcomes. These suggest that the potent mucosal IgA response underlies the observed cross-protection, aligning with previous studies that emphasized the relatively potent role of IgA compared to CD8^+^ T cells in intranasally vaccinated mice [56]. Unlike serum IgA, which predominantly exists in monomeric form, most IgA in external secretions is polymeric, a characteristic linked to enhanced cross-reactivity against heterologous strains [57,58,59,60]. The notable discrepancy between survival and viral shedding is also attributable to the IgA-related mechanism as well. While polymeric sIgA may not fully block virus entry during heterosubtypic infection, it can facilitate intracellular neutralization and tether newly budded virions on the surface of the infected cells, thereby limiting viral spread and reducing symptom severity [60,61,62,63]. The viral titers were measured at the peak of primary replication (3 dpi), before the IgA-mediated restriction became evident. Consistent with this timing, the rGD19/19PB2-vaccinated mice began to regain body weight from 4 dpi. Moreover, sIgA-mediated protection tends to avoid triggering strong inflammatory cascades, thus mitigating immunopathology and alleviating disease severity [64]. Together, these properties may explain why clinical manifestations are ameliorated in rGD19/19PB2-vaccinated mice during H1N1 and H3N2 challenge, even without a significant reduction in viral load. Previous studies also confirmed that secretory IgA modulates disease severity rather than achieving sterilizing immunity, permitting focal replication and shedding at subclinical levels [57]. In addition, the preferential elevation of IgA levels in BAL fluids, rather than in nasal washes, suggests that robust sIgA responses in the lower respiratory tract are crucial for mitigating excessive inflammation and improving clinical outcomes.

In addition to local humoral immunity, cellular immune response in the lungs may play an important role in cross-protection. Our prior work showed that BEI-inactivated recombinant clade 2.3.4.4b vaccines are internalized and their antigens are detected in the cytoplasm (manuscript in submission), suggesting that inactivated whole-virus vaccines might partially mimic live-virus infection and thereby induce T-cell responses. Notably, BEI inactivation has also been suggested to better preserve antigenic integrity and immunogenicity, further supporting its utility in eliciting robust cellular immunity [65]. Indeed, we noted a modest increase in IFN-γ-secreting cells among rGD19/19PB2-vaccinated mice (Figure S1). A comparative analysis of predicted CD8^+^ T-cell epitopes among GD19, Aichi/68, and PR8 (Table S1) revealed that PR8 and Aichi/68 share more epitopes in matrix 1 and, in particular, nucleoproteins (NPs) that are predicted to bind the MHC class I molecules expressed in BALB/c mice (H2-Dd, H2-Ld, H2-Kd). For NPs, the median predicted binding percentile was lower, indicating stronger predicted affinity. However, despite the role of NPs as a common target for cross-reactive T cells, our data (Figure 4C) indicate that, under our experimental conditions, both rGD19 and rGD19/19PB2 vaccines contained comparable amounts of NPs, in contrast to their marked difference in HA content [66]. Thus, the modestly enhanced T-cell response in the rGD19/19PB2 group may instead be directed toward epitopes in other segments, such as a single shared epitope in the cytoplasmic domains of NA from GD19 and Aichi/68 (Table S1). Given the scarcity of shared surface-glycoprotein epitopes, mucosal IgA rather than T-cell immunity is the principal contributor to the observed heterosubtypic protection.

Collectively, our findings suggest that the superior heterosubtypic protection conferred by rGD19/19PB2 is primarily driven by its higher HA content, which in turn boosts the mucosal sIgA response. As demonstrated by Okuya et al., broadly reactive, non-neutralizing HA-specific IgA can be instrumental in cross-protection [64]. Accordingly, the higher HA content in rGD19/19PB2 likely bolsters viral blockade at the point of entry and slows the spread of infection. While T-cell contributions appear modest, they may still complement sIgA in mitigating disease severity. Overall, the synergy between enhanced HA-specific IgA and supportive T-cell responses provides a plausible explanation for the pronounced protective efficacy of the intranasal rGD19/19PB2 vaccine. Future work should optimize the intranasal formulation by identifying non-irritating preservatives, selecting stabilizers that maintain HA potency, and pairing the vaccine with an accurate nasal spray device, thereby establishing this strain’s practical potential as an intranasal, inactivated influenza vaccine.

In this study, we successfully developed a highly productive pdm09 vaccine strain through the incorporation of cognate PB2 and demonstrated the protective efficacy of an inactivated whole-virion mucosal vaccine. In addition, this strain also showed lower murine virulence and better protection than conventional vaccine strains. As our study focused on single H1N1 strain using reverse genetics approach, additional studies are needed to confirm the broader applicability. Even so, our results may inspire further research toward developing more productive, safer, and potentially cross-protective mucosal vaccines and vaccination programs.

## 4. Materials and Methods

### 4.1. Eggs, Cells, and Viruses

Specific pathogen-free (SPF) eggs were purchased from VALO Biomedia (Osterholz-Scharmbeck, Lower Saxony, Germany), incubated at 37 °C for ten days, and used for the experiments. Madin-Darby canine kidney (MDCK) and 293T cells were acquired from the Korean Collection for Type Cultures (KCTC, Dajeon, Republic of Korea), and Calu-3 cells were purchased from the Korean Cell Line Bank (KCLB, Seoul, Republic of Korea). MDCK and 293T cells were maintained in Dulbecco’s Modified Eagle’s Medium (DMEM, Gibco, Waltham, MA, USA) supplemented with 10% Fetal Bovine Serum (FBS, Gibco), and Calu-3 cells were maintained in minimum essential medium with F12 and Glutamax (MEM/F12 + Glutamax, Gibco), supplemented with 10% FBS (Gibco).

Influenza A(H1N1) pandemic 2009 (pdm09)-like virus representing the WHO-recommended egg-based vaccine strain for the 2020–2021 northern hemisphere influenza season was provided by the National Culture Collection for Pathogens (NCCP, Cheongju, Republic of Korea) (NCCP 43400; A/Guangdong-Maonan/SWL1536/2019-like virus, GD19) [67]. The viruses were diluted and inoculated into 10-day-old SPF embryonated chicken eggs (ECEs). After incubating at 37 °C for three days, allantoic fluids were harvested, and those with positive hemagglutination (HA) titers were stored at −80 °C for further analysis.

### 4.2. Plasmids, Cloning, and Site-Directed Mutagenesis

The HA, NA, and PB2 genes of the GD19 virus were cloned into Hoffmann’s bidirectional transcription vector pHW2000 [68]. The inserted sequences were confirmed using the primers CMV-F and bGH-R. The six protein-coding genome segments of the A/Puerto Rico/8/1934 (PR8) virus (PB2, PB1, PA, NP, M, and NS) and the PB2 of the A/chicken/Korea/01310/2001 (H9N2) (01310) virus with I66M, I109V, and I133V mutations (310MVV) previously cloned into pHW2000 vector were used in this study [22,69]. In addition, by comparing coding sequences of 01310 PB2 and PR8 PB2 genes, we reversed the mammalian-adaptation markers of PR8 PB2 into a prototypic state, most of which represent avian-prevalent residues (K627E, S199A, N9D, T674A, A271T, and I588A) (detoxified PR8 PB2; dtxPB2). The site-directed mutagenesis of amino acid substitutions in the HA, NA, and PB2 genes was performed using the Muta-Direct Site-Directed Mutagenesis Kit following the manufacturer’s protocol, which was confirmed by sequencing using CMV-F and bGH-R primers (iNtRON Biotechnology, Sungnam, Republic of Korea).

### 4.3. Mini-Genome Assay

The effect of the variant PB2 genes on polymerase activities was measured using the pHW-NP-Luc plasmid as previously described [69]. A day before transfection, 293T cells were seeded in 12-well cell culture plates. The cells were co-transfected with 20 µg of the pHW-NP-Luc plasmid; PR8 PB1, PR8 PA, and PR8 NP genes cloned into the pHW2000 vector; and either 310MVV, dtxPB2, or GD19 PB2 genes. In addition, 0.1 µg of the Renilla luciferase plasmid pRL-TK (Promega, Madison, WI, USA) was co-transfected to serve as an internal control to normalize variations in transfection efficiency. The cells were harvested twenty-four hours after transfection, and luminescence was analyzed using the Dual-Glo Luciferase Assay System (Promega, Madison, WI, USA) following the manufacturer’s instructions. The luciferase activity was detected using a TECAN Infinite 200 Pro machine (Tecan Benelux bv, Giessen, The Netherlands), and the results were normalized to the activity of PR8 PB2.

### 4.4. Generation and Titration of the Recombinant H1N1 Viruses

The recombinant H1N1 viruses were generated using HA and NA genes derived from the GD19 virus. The remaining internal genes of the recombinant viruses were constituted with either six or five genomes from the PR8 virus, and the latter contained either PR8 PB2 with detoxifying mutations, 01310 PB2 with MVV mutations, or GD19 PB2. The viruses were generated using Hoffmann’s eight-plasmid DNA transfection system, as previously described [70]. Briefly, eight protein-coding genome segments cloned into a bidirectional pHW2000 vector were prepared. The 293T cells were seeded in six-well plates a day before transfection, and a mixture containing equal amounts of eight plasmids was transfected using Plus reagents and Lipofectamine 2000 (Life Technologies, Carlsbad, CA, USA) according to the manufacturer’s protocol. One milliliter of Opti-MEM (Life Technologies) and a 4 µg/well concentration of L-1-tosylamido-2-phenylethyl chloromethyl ketone (TPCK)-treated trypsin (Sigma-Aldrich, St. Louis, MO, USA) were added to the wells after 24 h. A day after, the supernatants were harvested, and 0.2 mL was used to inoculate 10-day-old SPF ECEs, which were subsequently incubated for three days at 37 °C.

The generation of recombinant viruses was confirmed by hemagglutination (HA) assay using 1% chicken red blood cells (RBCs). The sequences of the mutant viruses were confirmed via RT-PCR followed by Illumina MiSeq sequencing (Illumina, San Diego, CA, USA), which was performed by Bionics Co., Ltd. (Seoul, Republic of Korea), as described previously [71]. Viral titers were measured after the eggs were passaged at least two times. Harvested viruses were serially diluted from 10^−1^ to 10^−9^ in 10-fold increments, and each dilution was inoculated into four 10-day-old SPF ECEs or MDCK cells seeded in a 96-well plate. After incubation for three days at 37 °C, the 50% chicken embryo infectious dose (EID_50_) and 50% tissue culture infectious dose (TCID_50_) were calculated via the Spearman–Karber method [72].

### 4.5. RT-PCR, Sequencing, and Sequence Analysis

The full genome sequences of the propagated viruses were confirmed via Illumina Miseq sequencing (BIONICS Co., Seoul, Republic of Korea). Briefly, viral RNA was extracted from the allantoic fluids using the Patho Gene-spin DNA/RNA Extraction Kit (iNtRON Biotechnology, Sungnam, Republic of Korea), and amplified via RT-PCR with 0.2 μM MBTuni-12 and MBTuni-13 primers using the SuperScript IV one-step RT-PCR system (Invitrogen, Waltham, MA, USA). All eight vRNA segments were amplified simultaneously and confirmed by gel electrophoresis of the PCR products. The raw sequencing reads generated by the Illumina Miseq were analyzed using Geneious Prime (v.2023.2.1., Biomatters Ltd., Auckland, New Zealand). Paired, trimmed, and merged reads were assembled into contigs. The consensus sequences derived from the assembled contigs were subjected to BLAST analysis (https://blast.ncbi.nlm.nih.gov/, accessed on 10 Feburary 2024), which identified eight genomic segments of the influenza virus. The coding sequences of the HA and NA genes of the NCCP 43400 virus were confirmed to be identical to A/Guangdong-Maonan/SWL1536/2019 (H1N1) virus (GISAID ID; EPI_ISL_419003). The genome sequences of the reference vaccine strain during the 2009 pandemic H1N1 outbreak, A/California/04/2009 (EPI_ISL_29573), and during the 2020–2021 northern hemisphere influenza season, A/Guangdong-Maonan/SWL1536/2019 (EPI_ISL_377080), were obtained from the Global Initiative for Sharing All Influenza Data (GISAID) EpiFlu™ Database (https://gisaid.org/, accessed on 21 December 2024). The sequence comparison was performed using the BioEdit program (v7.2.5).

### 4.6. Growth Kinetics of the Recombinant H1N1 Viruses in Mammalian Cells

To evaluate the replication ability of the recombinant H1N1 viruses in mammalian cell lines, MDCK and Calu-3 were seeded in 12-well plates (5 × 10^5^ cells/mL) a day before the experiment. The cells were washed twice with PBS, and a 0.01 MOI of the virus diluted in DMEM [supplemented with 1% bovine serum albumin (BSA) (Fraction V) (MP Biomedicals), 20 mM HEPES, penicillin-streptomycin (Thermo Fisher Scientific, Waltham, MA, USA), and 1 µg/mL of TPCK-treated trypsin (Sigma-Aldrich, St. Louis, MO, USA)] was inoculated into MDCK and Calu-3 cells. After an hour of incubation, the inoculated virus was removed and washed twice with PBS followed by the addition of 1 mL of fresh medium. The inoculated cells were incubated for three days and the supernatants were harvested 0, 24, 48, and 72 h post-inoculation. The viral titer at each time point was measured using the 50% tissue culture infective dose (TCID_50_). The supernatants were diluted 10-fold and inoculated into MDCK cells prepared in 96-well plates. The viral titers were calculated via the Spearman–Karber method [72].

### 4.7. Analysis of HA Yield

To compare the antigen quantity of the vaccine strains in the same volume of allantoic fluid, 100 EID_50_/0.1 mL of each virus was inoculated into 10-day-old SPF ECEs and incubated for 72 h at 37 °C. The harvested allantoid fluids were centrifuged at 3000 rpm for 10 min at 4 °C to eliminate debris. Subsequently, the supernatants were partially purified by ultracentrifugation at 35,000 rpm for 1 h at 4 °C with a 30% sucrose cushion using a Type 70Ti rotor (Beckman Coulter, CA, USA). After resuspending the pellets in 500 μL of PBS, they were aliquoted and stored at −80 °C. The total protein content of virus concentrates was assessed using the BCA assay kit (Takara Bio Inc., Shiga, Japan) according to the manufacturer’s protocols. Samples were lysed using Laemmli SDS sample buffer (6×) and separated by SDS-PAGE using Bolt Bis-Tris Plus protein gels, 4–12% (Invitrogen), under reducing conditions. The viral protein bands were identified by Western blots using the PM2700 ExcelBand^TM^ 3-color Broad Range Protein Marker (SMOBIO Technology, Hsinchu, China). The immunostaining for the Western blots was performed using Influenza A NP Polyclonal antibody (PA5-32242, Invitrogen) or anti-influenza A virus H1N1 HA monoclonal antibody (MAB5637-M33, Abnova, Taipei City, Taiwan) as primary antibodies. The visualized bands were analyzed using ImageJ software (version 1.54p) [73].

### 4.8. Inactivated Vaccine Preparation

For the inactivation of the recombinant viruses, 0.1 M binary ethylenimine (BEI) solution (Sigma-Aldrich, St. Louis, MO, USA) was added to the allantoic fluids and incubated for 24 h at 37 °C followed by the addition of 1 M sodium thiosulfate to stop the reaction. The inactivated fluids were centrifuged at 3000 rpm for 10 min and supernatants were inoculated into 10-day-old SPF ECEs which were subsequently incubated for three days at 37 °C for the verification of inactivation. For the preparation of the vaccine for intramuscular inoculation, AddaVax™ (InvivoGen, San Diego, CA, USA) was used as an MF59-like adjuvant according to the manufacturer’s instructions.

### 4.9. Animal Experiments

To evaluate the mouse pathogenicity and efficacy of the vaccine strains, six-week-old female BALB/c mice were purchased from Orient Bio (Sungnam, Republic of Korea). All mice were anesthetized prior to vaccination and challenge via an intraperitoneal injection of Avertin (tribromoethanol, Sigma-Aldrich) (200 mg/kg).

For pathogenicity assessment, eight BALB/c mice per group were intranasally inoculated with either 10^6^ EID_50_/50 µL of each virus or an equal volume of phosphate-buffered saline (PBS). Mortality and weight loss were observed in five mice for 14 days, and mice that lost more than 20% of their initial weight were euthanized. To assess the replication efficiency of the virus in respiratory tracts, the lungs and nasal turbinates of three mice were collected 3 days post-inoculation and stored at −80 °C until use. The lungs were homogenized using a TissueLyzer 2 (Qiagen, Hilden, Germany) with 5 mm diameter stainless steel beads in a volume of PBS equal to 10% of the lung weight in suspension. Then, PBS was added to the homogenized tissue suspension to achieve a final 10-fold dilution. After centrifugation at 3000 rpm for 10 min, the supernatants were used to measure viral titers in MDCK cells.

For intramuscular vaccine assessment, fifteen mice per group were inoculated with 100 µL of BEI-inactivated, MF59-adjuvanted vaccines intramuscularly in each hindlimb. The allantoic fluids harvested after inoculating 100 EID_50_/0.1 mL of each virus were used to formulate the inactivated vaccine, which was formulated by mixing equal volumes of allantoic fluids and AddaVax™ (InvivoGen). For the negative control group, fifteen mice received an equal volume of PBS mixed with MF59 adjuvant. Serums were collected in five mice per group for three weeks at one-week intervals. For intranasal vaccine assessment, mice were intranasally inoculated with 50 µL of BEI-inactivated vaccines either once or twice at 2-week intervals. A period of 2 weeks after the final vaccination, mice were challenged with either 10 MLD_50_ of the homologous strain or 1 MLD_50_ of the heterologous strain. For five mice per group, mortality and weight loss were observed for 14 days, and mice that lost more than 20% of their initial weight were euthanized. For three mice per group, the lungs and nasal turbinates were harvested 3 days post-challenge to determine viral titers. Serum, nasal wash, and bronchoalveolar lavage (BAL) fluids were harvested in five mice per group 2 weeks after each vaccination prior to challenge for the evaluation of humoral and mucosal immune responses.

### 4.10. Hemagglutinin Inhibition (HI) Test

A hemagglutinin inhibition (HI) test was performed following WHO-recommended standard protocols [74]. Serum samples were treated with a receptor-destroying enzyme (RDE; Denka Seiken Co., Tokyo, Japan) for 18 h at 37 °C followed by incubation at 56 °C for 1 h in order to eliminate nonspecific reactions caused by proteins present in the serum. RDE-treated, heat-inactivated sera were serially diluted two-fold with PBS with a starting dilution of 1:8, and reacted with an equal volume of 4 [22] hemagglutination (HA) titers of challenged virus for 30 min. Then, 25 μL of 1% chicken RBCs was added, and the serum antibody titer was recorded after 40 min of incubation at 4 °C. The HI titer was defined by assessing the highest serum dilution exhibiting complete inhibition of hemagglutination.

### 4.11. Viral Neutralization (VN) Test

Each RDE-treated, heat-inactivated serum sample was serially diluted two-fold with DMEM in a 96-well plate with a starting dilution of 1:16 and incubated with the challenged virus diluted to 100 TCID_50_ per well for 1 h at 37 °C. After incubation, the mixture was overlayed on MDCK cells seeded in a 96-well plate a day before. The cells were then incubated at 37 °C under 5% CO_2_ for 3 days, and the results were analyzed by performing an HA test using the supernatants. The VN titer corresponded to the highest serum dilution that completely neutralized the infection as detected by the absence of hemagglutination.

### 4.12. Antigen-Specific Enzyme-Linked Immunosorbent Assay (ELISA)

The levels of IgG and IgA induced by the intranasal inoculation of whole-inactivated virus vaccines were evaluated via antigen-specific enzyme-linked immunosorbent assay (ELISA) using serum, nasal wash, and BAL fluids. Purified H1N1 and H3N2 antigens were diluted to 16 HAU/50 μL for IgG and 256 HAU/50 μL for IgA evaluation in coating buffer (0.1 M NaHCO_3_, 0.1 M Na_2_CO_3_), and 50 μL of diluted antigens was coated onto a 96-well immunoplate. After overnight incubation of the coated plate at 4 °C, the wells were washed three times with PBST (0.05% Tween20 in PBS) and blocked with 1% BSA in PBST to prevent nonspecific binding for 1 h at room temperature (RT). Then, the plates were incubated with two-fold-serially diluted serum, nasal wash, or BAL samples for 2 h and washed three times with PBST afterward. For the detection of IgG and IgA, plates were incubated with horse-radish peroxidase (HRP)-conjugated secondary anti-mouse IgG (Abcam, Cambridge, UK) or goat anti-mouse IgA (Invitrogen, Waltham, MA, USA) for 1 h at RT. After washing the plates three times, TMB substrate solution was added for HRP development, which was stopped with 0.1 M sulfuric acid (H_2_SO_4_) solutions. The optical density (OD) at 450 nm was detected using an ELISA reader (Tecan Benelux bv, Giessen, The Netherlands), and endpoint titers were calculated.

### 4.13. Flow Cytometric Analysis

In order to evaluate the cellular immune response following heterologous challenge in intranasally vaccinated mice, flow cytometric analysis was performed using lungs and spleens harvested 5 days post-challenge. For splenocyte isolation, the spleens were homogenized using 70 um cell strainers and 3 ml syringe plungers, and the pass-through was centrifuged at 5000× *g* for 5 min at room temperature. After the lysis of the red blood cells in the pellets using RBC lysing buffer (Sigma-Aldrich), the cells were further centrifuged and resuspended using complete RPMI medium. To isolate lung lymphocytes, dissected and homogenized lungs were incubated in digestion solution (0.1 mg/mL DNase 1 and 1 mg/mL collagenase D, Sigma-Aldrich) for 1 h at 37 and passed through 100 uL cell strainers using complete RPMI medium. After RBC lyses, the cells were centrifuged, and lymphocytes were collected from the density gradient layer between 44% and 66% Percoll solutions. After enumerating viable cells, the splenocytes and lung cells were diluted and seeded onto a 96-well cell culture plate and subsequently stimulated with purified H_3_N_2_ antigen for 12–14 h at 37 in a 5% CO_2_ incubator.

To analyze the T cell phenotypes, stimulated cells were initially stained with LIVE/DEAD Fixable Near-IR dye (Invitrogen, L34975) for live cell gating and subsequently stained with surface marker antibodies: anti-mouse CD3 (clone 17A2, APC), CD4 (clone GK1.5, FITC), and CD8a (clone 53-6.7, PE) monoclonal antibodies (eBioscience, San Diego, CA, USA). For intracellular cytokine staining (ICS), the cells were fixed, permeabilized, and stained with anti-mouse IFN-γ (AF700, BD Bioscience, San Jose, CA, USA) and TNF-α (PE/Dazzle, BioLegend, San Diego, CA, USA) antibodies. The stained cells were analyzed using SONY Cell Sorter (SH800S, SONY Biotechnology, San Jose, CA, USA) and FlowJo v10 software.

### 4.14. Statistical Analysis

Statistical significance was analyzed using GraphPad Prism software (version 10.0.2 for Windows, GraphPad Software, Boston, MA, USA). The viral titers, HA yields, protein band intensities, HI titers, SN titers, endpoint ELISA titers, and polymerase activities were compared via one-way ANOVA followed by Tukey’s multiple comparisons test. The growth kinetics analysis in mammalian cells was compared via two-way ANOVA, followed by Tukey’s multiple comparisons test as described in the figure legends. The results were considered statistically significant if *p* < 0.05.

### 4.15. Ethical Statement

All mouse experiments were carried out at the College of Pharmacy of Seoul National University (Seoul, Korea) following the recommendations of the National Institutes of Health’s Public Health Service Policy on the Humane Care and Use of Laboratory Animals (PHS Policy). The protocol was reviewed and approved by the Institutional Animal Care and Use Committee (IACUC) of Seoul National University (SNU-240820-4).

## Data Availability

The data presented in this study are available in this article (and Supplementary Material).

## Supplementary Materials

The supporting information can be downloaded at https://www.mdpi.com/article/10.3390/ijms26125489/s1.

## Abbreviations

The following abbreviations are used in this manuscript:

ECE	Embryonated chicken egg
Pdm09	Pandemic 2009
BEI	Binary ethylenimine
EID	Egg infectious dose
TCID	Tissue culture infectious dose
GD19	A/Guandong-Maonan/SWL1536/2019
