# Supplementary material for: A High-Yield Recombinant Inactivated Whole-Virion Nasal Influenza A(H1N1)pdm09 Virus Vaccine with an Attenuated PB2 Gene
Source: Int J Mol Sci. 2025 Jun 7;26(12):5489. doi: 10.3390/ijms26125489 (PMC12193355; doi:10.3390/ijms26125489)
Supplement: Supplementary file 1 [file ijms-26-05489-s001.zip › ijms-3619504-supplementary.pdf]

Table S1. Predicted T cell epitopes of GD19, Aichi/32, and PR8 viruses [a]

Segment	Epitope	Amino acid Position	BALB/c mouse MHC-1 Allele	Median Binding Percentile [b]
**NA** [c]	NPNQKIITI	2-10	H2-Ld	0.2
**M** [d]	LSPLTKGIL	52-62	H2-Dd	0.14
	SGPLKAEIA	17-25	H2-Dd	0.32
	LYRKLKREI	99-107	H2-Kd	0.38
	LGFVFTLTV	60-68	H2-Dd	0.4
	RGLQRRRFV	72-80	H2-Dd	0.6
	IAQRLEDVF	24-32	H2-Ld	0.67
	IYNRMGAVT	131-139	H2-Kd	0.72
	QMVTTTNPL	164-172	H2-Kd	0.84
**NP** [d]	FYIQMCTEL	39-47	H2-Kd	0.01
	AYERMCNIL	218-226	H2-Kd	0.04
	TYQRTRALV	147-155	H2-Kd	0.04
	RGINDRNFW	199-207	H2-Dd	0.22
	RLIQNSLTI	55-63	H2-Kd	0.26
	AGLTHMMIW	131-139	H2-Dd	0.3
	YGPAVASGY	281-289	H2-Dd	0.48
	FSVQRNLPF	412-420	H2-Dd	0.52
	MSNEGSYFF	481-489	H2-Ld	0.66
	HMMIWHSNL	135-143	H2-Ld	0.7
	KYLEEHPSA	77-85	H2-Kd	0.82
	ENPAHKSQL	320-328	H2-Dd	0.86
	MSNEGSYFF	481-489	H2-Dd	0.86

[a] Prediction parameters were 9-mer (peptide length) and H2-Kd, H2-Ld, and H2-Dd (MHC alleles); NetMHCpan 4.1 EL and NetMHCpan 4.1 BA were used as prediction models.

[b] A lower percentile indicates stronger binding or a higher probability of elution. Peptides with a percentile rank lower than or equal to 1% were included in the analysis, according to the predetermined threshold suggested by IEDB.

[c] Identical epitopes between GD19 and Aichi/68 viruses.

[d] Identical epitopes between Aichi/68 and PR8 viruses.

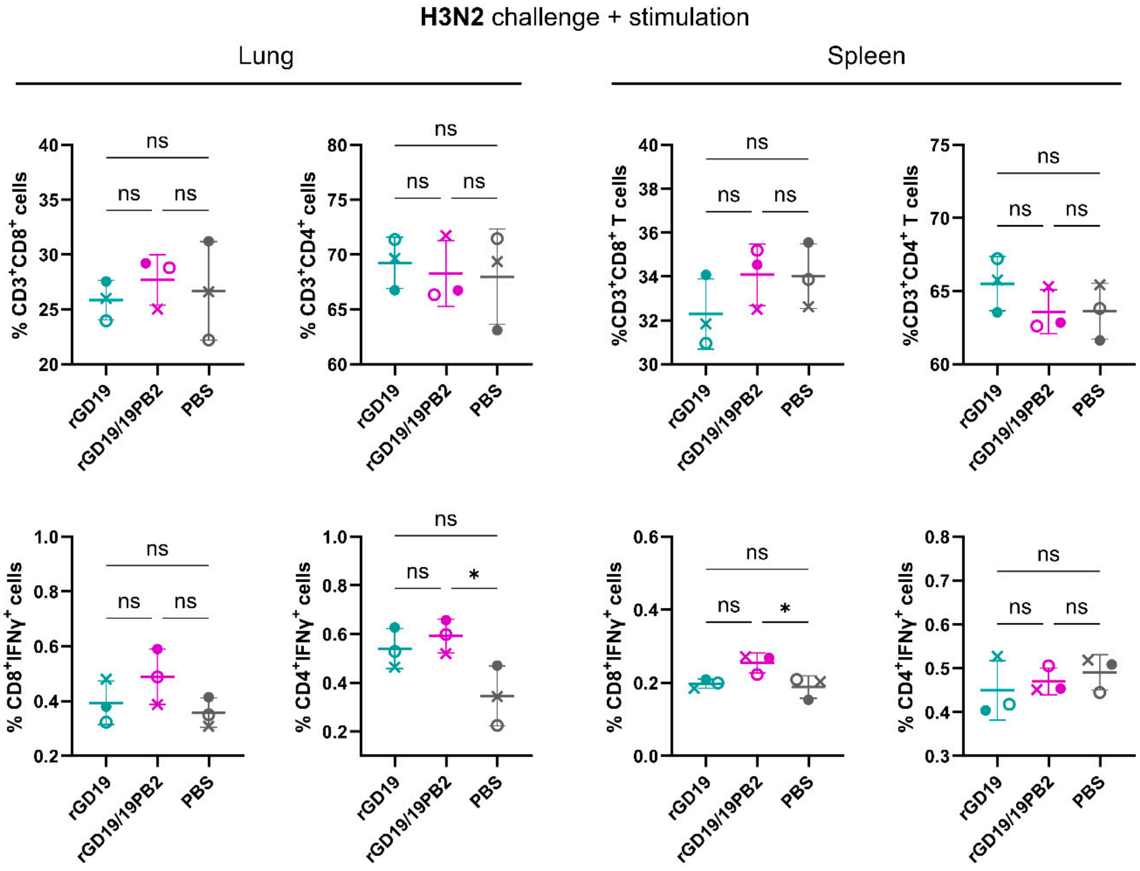

Figure S1. Flow cytometric analysis of lungs and spleens of mice following heterologous challenge. To assess the role of cellular immunity in heterosubtypic protection of rGD19/19PB2, lungs and spleens of mice vaccinated twice were harvested 5-days post challenge with heterologous virus (A/Aichi/68, H3N2) two weeks after receiving second vaccination (n=3/group). After the dissociation of splenocytes and lung cells, cells were incubated with purified A/Aichi/68 (H3N2) virus for 12-14 hours and intracellular staining was performed to detect IFN-γ- secreting cells. The cells were gated as follows; lymphocytes - single cells - live cells - $CD3^+$ - $CD8^+$ or $CD4^+$ - IFN-γ+ cells. Gating and population analysis were performed using FlowJo software. The statistical significances were analyzed by one-way ANOVA followed by Tukey's multiple comparisons test.

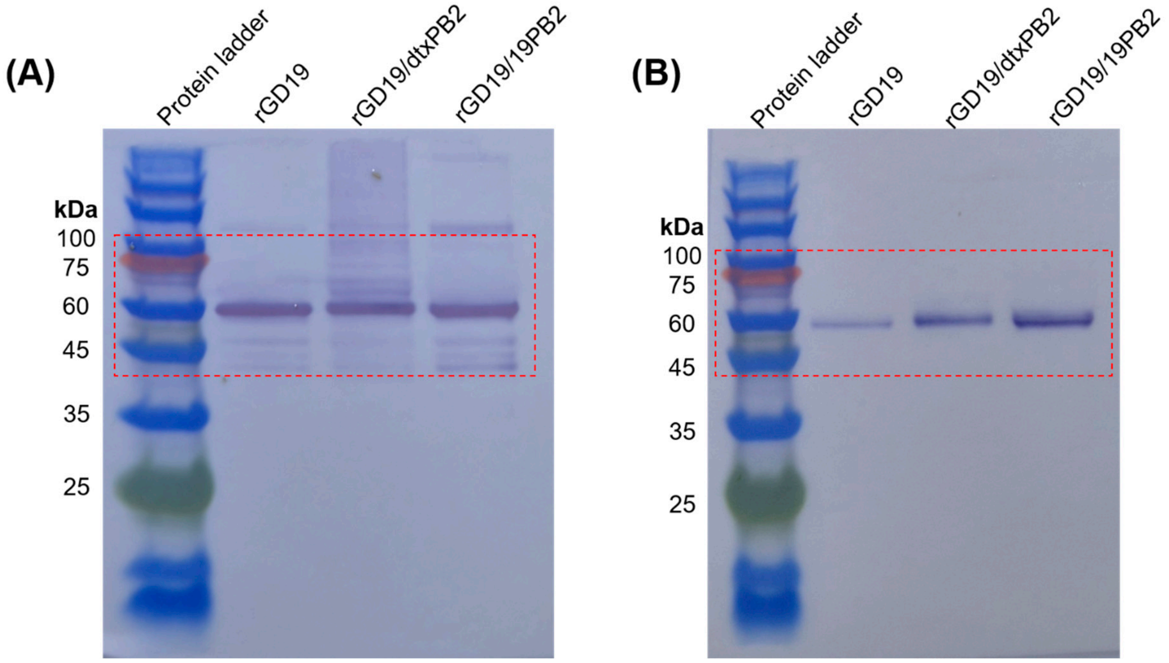

Figure S2. Uncropped, raw image corresponding to Figure 3C. (A) Visualization of bands corresponding to Influenza NP. (B) Visualization of bands corresponding to H1N1 HA. The red dashed box indicates the cropped region used in the final figure. Molecular weight markers are included and labeled as "protein ladder" above the image.
